# Consumption patterns of meat, poultry, and fish after disaggregation of mixed dishes: secondary analysis of the Australian National Nutrition and Physical Activity Survey 2011–12

**DOI:** 10.1186/s40795-017-0171-1

**Published:** 2017-07-01

**Authors:** Zhixian Sui, David Raubenheimer, Anna Rangan

**Affiliations:** 1 Charles Perkins Centre, School of Life and Environmental Sciences, University of Sydney, Camperdown, NSW 2006 Australia; 2 Nutrition and Dietetics Group, School of Life and Environmental Sciences, The University of Sydney, Camperdown, NSW 2006 Australia

**Keywords:** Meat, Nutrients, National nutrition survey, Food disaggregation, Food consumption

## Abstract

**Background:**

With the increased consumption of mixed dishes, the need for more precise quantitative data on individual food components is becoming more important. This paper aims to examine the consumption of meat, poultry, and fish before and after disaggregation of mixed dishes, and its contribution to energy and nutrient intakes in a representative sample of Australians.

**Methods:**

This study utilised a 24-h recall of 12,153 people aged two years and over participating in the 2011–12 National Nutrition and Physical Activity Survey. Consumption of meat/poultry/fish was examined before and after disaggregation of mixed dishes where all meat products and mixed dishes were separated into individual food components. Comparison between these two methods was undertaken for consumption data and contribution to energy and nutrient intakes, reported by meat type.

**Results:**

Disaggregation of mixed dishes resulted in lower estimated intakes of red meat (9%), poultry (25%), and fish (18%) but higher estimates of processed meat (17%). Meat/poultry/fish contributed approximately 25% of total energy intake, 49% protein, 29% saturated fat, 26% iron, and 38% of zinc intake after disaggregation, which was significantly higher than their contributions reflected in survey data containing mixed dishes. Per-capita consumption of all meat/poultry/fish was 118 g/day for children and 162 g/day for adults, with chicken and beef being the highest contributors.

**Conclusion:**

These findings provide a detailed picture of meat/poultry/fish consumption in Australia, and emphasise the need for population studies to disaggregate reported food information to provide a more precise estimate of consumption.

**Electronic supplementary material:**

The online version of this article (doi:10.1186/s40795-017-0171-1) contains supplementary material, which is available to authorized users.

## Background

Interest in meat consumption and its effect on health has grown tremendously over the past few years [1–5]. In Australia, meat, poultry, fish and alternatives’ is one of the five core food groups, part of a balanced diet [6] and provides key nutrients such as protein, long-chain omega 3 fatty acids, vitamin B12, iron and zinc [7]. Although it is a diverse food group, comprising fresh meat, processed meat, fish, legumes and other alternatives, recommended intakes for this food group have been set for population subgroups, depending on age, gender and life stage [6, 7]. These recommendations are based on extensive food modelling, taking into account the type of meat, nutrient contributions as well as upper limits to reduce the risk of chronic disease [7, 8].

In order to estimate meat consumption, detailed information is required on intakes of meat consumed as individual foods as well as recipe information for mixed dishes and meat products. For example, meat-based mixed dishes such as stir-fries and curries, typically contain meat, sauce, vegetables and/or cereals such as rice or pasta. Consumption data may be reported in broad food groupings, for example as in the first release of the national nutrition survey results [9], or as disaggregated into individual food components and meat types. Due to the increasingly wider variety of mixed dishes and meat products available [10, 11], disaggregation of these dishes into their component parts is becoming more important, and provides a more precise estimate of meat intake.

In this study, a detailed analysis of meat/poultry/fish consumption is undertaken using the 2011–12 National Nutrition and Physical Activity Survey of Australia (NNPAS), the most recent nationally representative survey and the first since 1995 covering both children and adults. All meat, poultry, fish and processed meat consumed were examined by disaggregating all meat products and mixed dishes into individual meat types and other food components. The aim of this study was to compare the impact of disaggregation on population meat/poultry/fish intake levels and nutrient contribution, as well as an assessment of the consumption of disaggregated meat/poultry/fish types according to gender, age group, and socio-economic status, factors known to affect consumption [9, 12–15]. These findings will provide a more precise estimate of meat/poultry/fish consumption and help to inform evidence-based dietary advice.

## Methods

### Subjects and dietary data collection

This study utilized the 2011–12 National Nutrition and Physical Activity Survey (NNPAS), undertaken by the Australian Bureau of Statistics (ABS) between May 2011 and June 2012. Ethics approval for the survey was granted by the Australian Government Department of Health and Ageing Departmental Ethics Committee in 2011 [16].

The NNPAS survey provided food and nutrient information from 24-h recalls and information on selected dietary behaviours by age group and gender at the national level. To take account of possible seasonal effects on health and nutrition characteristics, the NNPAS sample was spread across a 12-month enumeration period. The survey covered a sample of approximately 9500 private dwellings across Australia. Further details about the scope and the methodology of the survey are available from the ABS [16]. A total of 12,153 respondents were interviewed face-to-face for the collection of dietary intake data using a 24-h recall. The recall process followed the 5-step Automated Multiple-Pass Method which navigates the interviewer through the recall, posing standardized questions and providing response options for different foods and beverages [17]*.* The interviewers used a food model booklet with sample pictures and measurements to assist the respondents with describing the amount of foods and beverages consumed [16]. A second 24-h recall was collected from a subsample but only data from the first recall were used for this secondary analysis, with the results weighted to reflect the Australian population [16].

### Classification of meat/poultry/fish

#### Survey classification (i.e. before disaggregation)

The first release of nutrition data from the NNPAS survey reported food and nutrition intake data based on the food classification system developed by Food Standards Australia and New Zealand (FSANZ) [16]. The system categorises food into food groups with hierarchical levels as major, sub-major, minor, and sub-minor groups. The major food group for meat/poultry was ‘meat, poultry and game products and dishes’ and for fish ‘fish and seafood products and dishes’ , which included individually recorded meat/poultry/fish items as well as meat/poultry/fish products and dishes where meat/poultry/fish was a major component of the dish (i.e. the greatest component by weight in the recipe) [9]. For dishes where meat/poultry/fish was a minor component (e.g. pie, lasagne or pizza where grains/cereals were the greatest component by weight in the recipe), the consumption of meat/poultry/fish was not captured and the weight and the nutrients of meat/poultry/fish in these dishes were counted under the other major food groups (e.g. ‘cereal-based products and dishes’). Total meat/poultry/fish consumption comprised all individually recorded items and mixed dishes where the meat/poultry/fish was a major component (but includes other food components from the mixed dishes, and excludes meat/poultry/fish from dishes where meat/poultry/fish was a minor component).

#### Disaggregated classification

In order to capture all meat/poultry/fish consumed on the day prior to the interview, all meat products and mixed dishes were disaggregated into individual components using the AUSNUT 2011–13 recipe file [18]. The recipe file is based on information found in common Australian cook books and recipe websites, known commercial kitchen preparation procedures and product preparation instructions, gross composition data, and cooking and preparation practices observed during the survey time period [19]. This method has been applied in previous analyses in the UK and Ireland to capture a more precise estimation of meat consumption [20, 21]. All meat/poultry/fish reported from ‘mixed dishes where meat/poultry/fish was a major or minor component’ were disaggregated (*n* = 1545). For example, the proportion of chicken in a green chicken curry was determined from the AUSNUT 2011–13 recipe file, and translated into grams of chicken consumed per participant [11]. Other examples included the amounts of ham and bacon consumed from pizzas or quiches, or the amount of fish in fish fingers. This process is comparable to that used internationally for similar purposes [22]. Total meat/poultry/fish consumption comprised all individually recorded items and individual meat/poultry/fish components from mixed dishes (as a major or minor component).

#### Meat types

The term ‘meat/poultry/fish’ as used in this study refers to all red meat, poultry, fish/seafood, organ/offal meat and processed meat but excludes any meat alternatives such as eggs, tofu, nuts and seeds, legume and beans. ‘Red meat’ refers to mammalian meat: beef (including veal), lamb (including mutton), pork, kangaroo, and game meat (including goat, venison, and rabbit) excluding organ/offal meat as described in the Australian Guidelines of Healthy Eating (AGHE) [6]. ‘Poultry’ refers to avian meat: chicken, turkey, duck and other poultry. The term ‘fish/seafood’ refers to all fresh/frozen finfish, seafood (molluscs and crustacea), canned fish and fish/seafood products (e.g. fermented, smoked and dried fish) [23]. All organ/offal meat were reported together because of the low frequency of consumption. ‘Processed meat’ included sausages (e.g. beef, pork and BBQ sausages), bacon, ham, salami, processed luncheon meat (e.g. devon, smoked chicken), and other processed meat (e.g. frankfurters, spam, beef jerky) [24]. Additional file 1: Table S1 includes a detailed description of all meat/poultry/fish types and related products under each meat/poultry/fish category.

### Nutrient contribution

The contribution to energy and nutrient intake was calculated for total meat/poultry/fish intake and by individual categories of meat/poultry/fish for each participant and then averaged across the relevant age/gender groups. The key nutrients examined included protein, total fat, saturated fat, long-chain omega 3 fatty acids, iron and zinc.

### Data and statistical analysis

Total meat consumption was calculated before disaggregation (using the survey classification) and after disaggregation, and per-capita intakes were compared using paired sample t-tests. Descriptive statistics were used to report the proportion consuming, per-capita *(average intake among all respondents)* and per-consumer intakes *(average intake among consumers only)* of total and individual meat, poultry and fish categories. Per-capita intake was reported as mean and standard deviation (SD) expressed as gram/day. Median intake, 25th and 75th percentiles were reported for per-consumer consumption. Chi-square, ANOVA or non-parametric (Kruskal-Wallis) tests were performed where appropriate to test for associations between meat intake and gender, age and socio-economic categories.

Meat/poultry/fish consumption analyses were reported according to gender, age group as defined by NNPAS (children: 2–3, 4–8, 9–13, 14–18 years; and adults: 19–30, 31–50, 51–70, and older than 70 years) and socio-economic categories (based on the Socio-Economic Index of Disadvantage for Areas (SEIFA), where the first SEIFA quintile indicates the most disadvantaged areas) [9]. Statistical analyses were performed using SPSS for Windows 22.0 software (SPSS Inc., Chicago, IL, USA). For all tests, a *P*-value of <0.05 was considered statistically significant.

## Results

### Effect of disaggregation of meat/poultry/fish from meat products and mixed dishes

Table 1 compares mean daily intakes of meat/poultry/fish consumption using consumption as reported using the survey classifications to that estimated when all mixed dishes are disaggregated on the basis of their ingredients, and reveals an 11.0% difference in total consumption. After disaggregation, meat/poultry/fish intakes were lower than those reported using the broader survey classifications. Daily intakes of red meat were 9.1% lower, poultry 25.3% lower, and fish/seafood 17.6% lower when disaggregated, whereas intake of processed meat was 17.4% higher using the disaggregated method.

**Table 1:** Per-capita consumption of meat/poultry/fish (g/day) – comparison before and after disaggregation of mixed dishes

	Before disaggregation mean (SD)^a^	After disaggregation mean (SD)^b^	Difference (%)
Red meat	62.2 (90.4)	57.0 (88.4)	9.1*
Beef, cut or mince	18.7 (77.3)	17.8 (58.0)	
Mixed dishes where beef is the major component	22.0 (78.0)	10.2 (43.5)	
Mixed dishes where beef is the minor component^c^	-	12.0 (32.0)	
Lamb, cut or mince	7.2 (37.0)	7.2 (37.0)	
Mixed dishes where lamb is the major component	4.9 (20.3)	1.8 (18.0)	
Mixed dishes where lamb is the minor component^c^	-	0.6 (8.2)	
Pork, cut or mince	6.0 (30.1)	5.1 (31.1)	
Mixed dishes where pork is the major component	2.6 (24.7)	1.1 (13.2)	
Mixed dishes where pork is the minor component^c^	-	0.8 (7.0)	
Kangaroo, cut or mince	0.2 (21.2)	0.2 (15.0)	
Game meat, cut or mince	0.1 (14.1)	0.1 (6.9)	
Poultry	57.9 (96.0)	46.2 (86.7)	25.3*
Chicken, cut or mince	24.3 (86.7)	22.7 (69.5)	
Other poultry, cut or mince	1.3 (12.3)	1.1 (18.1)	
Mixed dishes where poultry is the major component	32.3 (45.1)	16.2 (53.7)	
Mixed dishes where poultry is the minor component^c^	-	6.2 (7.3)	
Fish/seafood	26.0 (62.2)	22.1 (60.5)	17.6*
Finfish	6.5 (36.7)	6.5 (36.7)	
Crustacea and molluscs	1.3 (17.8)	1.3 (17.8)	
Packed fish and seafood	4.8 (23.7)	4.6 (23.7)	
Fish and seafood products	7.7 (32.3)	5.5 (32.3)	
Mixed dishes with fish or seafood as the major component	5.3 (18.6)	2.0 (18.6)	
Mixed dishes with fish or seafood as the minor component^c^	-	1.8 (19.5)	
Processed meat	21.9 (87.5)	26.5 (58.0)	−17.4*
Sausages, frankfurts and saveloy	10.2 (45.7)	10.2 (45.7)	
Bacon	2.8 (23.0)	2.8 (23.0)	
Ham	3.4 (19.9)	3.4 (19.9)	
Fermented, comminuted meats	1.2 (11.5)	1.2 (11.5)	
Processed delicatessen meat	3.1 (23.0)	3.1 (23.0)	
Mixed dishes where processed meat is the major component	0.5 (5.9)	0.3 (5.9)	
Mixed dishes where processed meat is the minor component^c^	-	4.8 (11.0)	
Total meat/poultry/fish	168.2 (189.4)	152.0 (128.9)	11.0*

*P-value <0.05 from independent t-test

^a^Values refer to the mass of all individually recorded items, and the total mass of mixed dishes where meat/poultry/fish was a major component but excludes mass from dishes where meat/poultry/fish was a minor component

^b^Values refer to the mass of the meat/poultry/fish components from all individually recorded items and from mixed dishes where meat/poultry/fish was a major or minor component

^c^Mixed dishes where meat/poultry/fish is the minor component covers foods such as pies, pastries, pizzas, quiches, soups and salads

### Disaggregated classification results: Proportion consuming

After disaggregation of all meat products and mixed dishes, approximately 92.6% males and 90.1% females reported consuming some meat/poultry/fish on the day surveyed (Table 2) with the gender difference being statistically significant (*P* < 0.01). The proportion of meat/poultry/fish consumption was 90.4% for children and 91.5% for adults. Red meat was consumed by 48.6% of participants, with beef as the most frequently reported type (males 41.8%, females 34.7%). Poultry was consumed by 37.7% of participants mainly as chicken (males 36.8%, females 36.9%). Fish/seafood was consumed by 21.4% of participants (finfish 9.7%, seafood 5.4%, canned fish 7.8%, and fish/seafood products 1.6%). Processed meat was consumed by 37.8% of participants, with higher frequencies reported by males than females (41.4% versus 34.6%, *P* < 0.01). The most frequently reported types of processed meat consumed were ham (males 19.4%, females 16.8%), bacon (males 15.3%, females 12.4%), and sausage (males 8.5%, females 5.8%).

**Table 2:** Proportion of persons consuming meat/poultry/fish by gender for children and adults after disaggregation of mixed dishes

Proportion (%)	Total	Total male	Total female	Children			Adults		
				Total	Male	Female	Total	Male	Female
Red meat	48.6	52.4	45.2^^^	46.0	47.4	44.6^^^	49.4	54.1	45.4^^^
Beef	38.0	41.8	34.7^^^	38.3	39.9	36.6^^^	38.0	42.4	34.2^^^
Lamb	8.1	8.6	7.6^^^	6.1	6.6	5.6	8.7	9.3	8.2^^^
Pork	7.5	8.0	7.0	5.6	5.4	5.9	8.0	8.9	7.3^^^
Kangaroo	0.3	0.4	0.3	0.4	0.4	0.4	0.3	0.4	0.3
Game meat	0.1	0.1	0.1	0.0	0.0	0.1	0.1	0.2	0.1
Poultry	37.7	37.7	37.7	38.4	37.5	39.4	37.5	37.8	37.2^^^
Chicken	36.8	36.8	36.9	38.0	37.1	38.9	36.5	36.6	36.4
Other	1.3	1.4	1.1	0.7	0.6	0.9	1.4	1.6	1.2
Organ/offal meat	0.1	0.2	0.1	0.1	0.1	0.0	0.1	0.2	0.1
Fish/seafood	21.4	20.3	22.4^^^	14.3	13.7	14.9	23.5	22.5	24.4^^^
Finfish	9.7	10.0	9.4	7.1	6.9	7.3	10.4	11.0	10.0
Seafood	5.4	5.1	5.7	3.1	3.0	3.2	6.1	5.8	6.3
Canned fish	7.8	7.1	8.5^^^	4.6	4.2	5.0	8.8	8.0	9.5^^^
Fish/seafood products	1.6	1.4	1.7	0.7	0.5	0.9	1.8	1.6	2.0
Processed meat	37.8	41.4	34.6^^^	42.9	44.7	41.1^^^	36.2	40.4	32.8^^^
Sausage	7.1	8.5	5.8^^^	9.0	9.9	8.2^^^	6.5	8.1	5.1^^^
Ham	18.0	19.4	16.8^^^	21.1	22.1	20.0^^^	17.1	18.6	15.9^^^
Bacon	13.8	15.3	12.4^^^	14.3	15.4	13.1^^^	13.6	15.3	12.2^^^
Salami	5.7	6.7	4.8^^^	6.3	7.3	5.2^^^	5.5	6.4	4.6^^^
Luncheon meat	3.7	4.2	3.4	4.0	4.1	3.9	3.7	4.3	3.3
Other	1.9	2.2	1.5	3.3	4.3	2.2^^^	1.4	1.5	1.3
Total meat/poultry/fish	91.3	92.6	90.1^^^	90.4	90.8	90.0^^^	91.5	93.2	90.1^^^

^^^ *P*-value <0.05 for gender difference from Chi-square test

### Disaggregated classification results: Per-capita intake

**Fig. 1:**
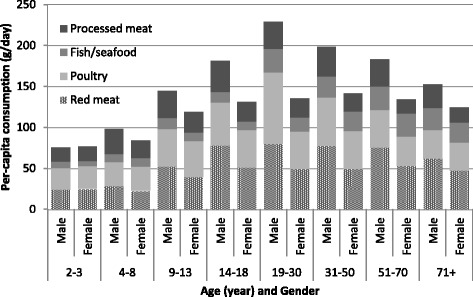
Per-capita consumption of meat/poultry/fish by age group and gender after disaggregation of mixed dishes, g/day

The meat/poultry/fish type with the highest per-capita intake was chicken (males 50.8 g/day, females 39.2 g/day), followed by beef (males 48.0 g/day, females 33.0 g/day), finfish (males 12.3 g/day, females 9.7 g/day), sausage (males 13.4 g/day, females 7.4 g/day), and lamb (males 11.7 g/day, females 7.7 g/day) (Table 3). The lowest intake per-capita was game meats and organ meats (<1.0 g/day). The mean per-capita intake of all meat/poultry/fish types was greater for males than females (*P* < 0.01), except canned fish and fish/seafood products. Adult males aged between 19 and 30 years were the highest consumers of meat/poultry/fish, and males aged 14–70 years were the highest red meat consumers (Fig. 1).

**Table 3:** Per-capita consumption of meat/poultry/fish by gender for children and adults after disaggregation of mixed dishes (g/day)

Mean (SD) (g)	Total	Total male	Total female	Children			Adult		
				Total	Male	Female	Total	Male	Female
Red meat	57.0 (88.4)	68.5 (100)	46.9 (75.1)^^^	41.9 (69.3)	48.4 (79.1)	35.3 (56.9)^^^	61.6 (92.8)	75.2 (105.2)	50.1 (79.1)^^^
Beef	40.0 (75.8)	48.0 (85)	33.0 (65.9)^^^	31.4 (59.7)	36.3 (67.7)	26.3 (49.8)^^^	42.6 (79.8)	51.9 (89.6)	34.8 (69.5)^^^
Lamb	9.6 (40.2)	11.7 (47.4)	7.7 (32.3)^^^	6.3 (31.9)	7.5 (36.4)	5.1 (26.5)^^^	10.5 (42.3)	13.0 (50.5)	8.5 (33.7)^^^
Pork	7.0 (33.7)	8.4 (38.2)	5.8 (29.2)^^^	4.1 (23.2)	4.4 (26.4)	3.8 (19.5)^^^	7.9 (36.3)	9.7 (41.2)	6.4 (31.4)^^^
Kangaroo	0.3 (8.8)	0.3 (8.3)	0.2 (9.3)	0.1 (3.3)	0.1 (4.3)	0.0 (1.6)	0.3 (9.9)	0.4 (9.3)	0.3 (10.4)
Game meat	0.1 (3.9)	0.2 (4.9)	0.1 (2.7)	0.0 (1.2)	0.0 (0.0)	0.0 (1.7)	0.1 (4.4)	0.2 (5.6)	0.1 (3.0)
Poultry	46.2 (86.7)	52.7 (100)	40.5 (72.5)^^^	38.9 (70.0)	40.0 (72.2)	37.8 (67.7)^^^	48.4 (91.0)	56.9 (107.3)	41.2 (73.8)^^^
Chicken	44.7 (84.8)	50.8 (97.9)	39.2 (70.8)^^^	38.2 (68.5)	39.4 (71.7)	36.9 (65.1)^^^	46.7 (89.0)	54.6 (104.9)	39.9 (72.3)^^^
Other	1.5 (19.4)	1.9 (22.5)	1.2 (16.3)^^^	0.7 (11.6)	0.6 (8.9)	0.9 (13.7)	1.7 (21.2)	2.3 (25.4)	1.3 (16.9)^^^
Organ/offal meat	0.0 (1.1)	0.0 (1.3)	0.0 (0.9)	0.0 (1.1)	0.0 (1.5)	0.0 (0.0)	0.0 (1.1)	0.0 (1.2)	0.0 (1.0)
Fish/seafood	22.1 (60.5)	23.3 (65.1)	21.1 (56.2)^^^	10.4 (33.6)	11.3 (36.9)	9.6 (29.8)^^^	25.6 (66.2)	27.3 (71.6)	24.2 (61.1)^^^
Finfish	10.9 (43.1)	12.3 (46.6)	9.7 (39.6)^^^	5.5 (25.1)	6.2 (28.6)	4.9 (20.9)^^^	12.6 (47.0)	14.3 (51.1)	11.0 (43.3)^^^
Seafood	3.5 (21.2)	3.5 (22.1)	3.5 (20.3)	1.4 (12.3)	1.4 (12.9)	1.4 (11.7)	4.2 (23.1)	4.3 (24.4)	4.1 (22.0)
Canned fish	5.9 (25)	5.7 (24.6)	6.0 (25.3)	2.9 (16.1)	3.2 (18.2)	2.5 (13.6)^^^	6.8 (27.0)	6.5 (26.4)	7.0 (27.6)^^^
Fish/seafood products	1.8 (25.1)	1.7 (28.3)	1.8 (21.8)	0.6 (9.5)	0.4 (6.6)	0.8 (11.6)	2.1 (28.1)	2.2 (32.4)	2.1 (23.9)
Processed meat	26.5 (58)	33.2 (67.3)	20.6 (47.5)^^^	26.9 (53.4)	31.2 (59.9)	22.5 (45.3)^^^	26.4 (59.3)	33.8 (69.5)	20.1 (48.1)^^^
Sausage	10.2 (43.4)	13.4 (51.6)	7.4 (34.4)^^^	11.0 (41.5)	13.5 (48.3)	8.5 (32.9)^^^	10.0 (43.9)	13.4 (52.6)	7.1 (34.8)^^^
Ham	5.0 (15.3)	5.8 (16.2)	4.2 (14.3)^^^	5.5 (16.3)	5.9 (15.3)	5.0 (17.2)^^^	4.8 (15)	5.7 (16.5)	4.0 (13.4)^^^
Bacon	4.7 (16.5)	5.9 (19.0)	3.7 (13.9)^^^	4.2 (15.3)	4.5 (14.9)	3.8 (15.6)^^^	4.9 (16.9)	6.4 (20.2)	3.7 (13.3)^^^
Salami	1.8 (13.1)	2.2 (14.0)	1.4 (12.3)^^^	1.3 (8.6)	1.6 (10.3)	1.0 (6.4)^^^	1.9 (14.2)	2.4 (15.0)	1.5 (13.4)^^^
Luncheon meat	3.0 (19.3)	3.3 (19.6)	2.7 (19.0)^^^	2.4 (15.2)	2.3 (13.7)	2.4 (16.6)^^^	3.2 (20.4)	3.6 (21.2)	2.8 (19.6)^^^
Other	1.8 (10.3)	2.6 (12.2)	1.2 (8.2)^^^	2.5 (13.7)	3.4 (16.0)	1.8 (10.9)^^^	1.6 (8.9)	2.3 (10.6)	1.0 (7.3)^^^
Total meat/poultry/fish	152.0 (128.9)	177.8 (146.8)	129.1 (105.6)^^^	118.2 (105.6)	131.0 (119.6)	105.2 (87.4)^^^	162.1 (133.5)	193.3 (151.6)	135.7 (109.2)^^^

^^^ *P*-value < 0.05 for gender difference from t-test

For children (Fig. 1), the per-capita total meat/poultry/fish consumption increased with increasing age (*P* < 0.01). For adults, the per-capita total meat/poultry/fish intake decreased with advancing age (*P* < 0.01), due mostly to a decrease in the consumption of poultry (*P* < 0.01).

Per-capita intake of processed meat was 26.9 g/day for children (approximately 22.8% of total consumption of meat/poultry/fish) and 26.4 g/day for adults (approximately 16.3% of total consumption of meat/poultry/fish) (Table 3). The per-capita consumption of processed meat increased with age among children (*P* < 0.01) but remained unchanged for adults (Fig. 1).

No significant differences were observed in the per-capita intakes of fish/seafood by gender or age category. When analyzed according to SEIFA quintiles, fish/seafood intake increased with socio-economic status, for both males (*P* < 0.01) and females (*P* < 0.01) (data in Additional file 1: Table S2).

### Disaggregated classification results: Per-consumer intake

The meat/poultry/fish type with the largest per-consumer intake was lamb (males 124.0 g/day, females 95.5 g/day), followed by sausage (males 151.5 g/day, females 101.0 g/day) and finfish (males 106.4 g/day, females 92.5 g/day) (Table 4). The smallest per-consumer intakes were for ham (17.0 g/day) and salami (18.5 g/day).

**Table 4:** Per consumer intake of meat/poultry/fish by gender for children and adults, median (25th and 75th percentile) after disaggregation of mixed dishes, g/day^a^

Median (25th – 75th percentile)	Total	Total male	Total female^^^	Children			Adults		
				Total	Male	Female	Total	Male	Female^^^
Red meat	98.1 (47.5–163.5)	107.0 (52.3–181.9)	91.0(43.8–150.0)	70.8 (34.7–131.2)	78.4 (38.8–147.0)	64.0 (29.2–115.9)	104.0 (54.0–169.0)	119.4 (59.9–189.7)	97.0 (49.0–156.0)
Beef	83.3 (36.3–155.3)	93.8 (40.8–166.2)	76.5(33.2–146)	62.8 (28.4–116)	66.0 (33.5–131.2)	52.8 (23.5–104.5)	94.2 (40.8–164.5)	99.0 (42.1–174.0)	83.3 (36.7–152.0)
Lamb	104.0 (61.0–156.0)	124.0 (68.6–171.7)	95.5(52.8–142)	96.5 (44.5–155.8)	103.1 (47.7–156.0)	72.8 (39.6–142.0)	104.0 (65.1–156.0)	130.3 (73.4–176.1)	99.0 (59.8–143.5)
Pork	75.0 (30.5–120.0)	80.0 (36.0–148.0)	73.8 (24.5–109.2)	51.7 (23.6–107.4)	56.4 (22.9–107.6)	42.2 (23.6–107.4)	82.0 (35.4–125.0)	89.8 (39.2–154.0)	75.0 (26.2–109.2)
Poultry	95.0 (57.0–166.0)	108.0 (60.3–184.2)	86.6 (51.2–143)	80.0 (46.8–129.8)	81.4 (50.0–138.7)	74.1 (42.5–114.0)	100.0 (60.0–176.2)	116.9 (66.0–195.7)	89.3 (55.8–149.0)
Chicken	93.6 (57.0–162.7)	107.5 (60.3–184.2)	85.9 (50.9–142.5)	80.0 (46.8–129.5)	81.4 (48.0–138.7)	74.1 (42.6–114.0)	100.0 (59.8–175.2)	116.6 (65.8–191.1)	89.3 (55.6–147.0)
Other	92.3 (33.6–151.9)	98.0 (70.4–166.0)	80.1 (23.1–143)	63.6 (27.7–150.0)	109.5 (58.1–158.0)	52.0 (27.7–84.8)	93.0 (36.0–153.8)	95.1 (70.4–166.0)	92.3 (17.6–143.0)
Fish/seafood	81.9 (46.1–126.8)	92.5 (51.4–144.0)	77.5 (41.6–117.3)	61.5 (30.0–101.3)	67.0 (33.0–117.8)	53.7 (26.0–86.3)	86.1 (49.0–133.3)	95.5 (61.1–152.5)	80.0 (46.1–121.6)
Finfish	103.5 (53.7–136.8)	106.4 (62.4–156.2)	92.5 (46.5–123.1)	62.7 (38.7–107.0)	85.9 (41.0–117.8)	58.0 (33.5–96.8)	104.5 (62.4–150.0)	110.0 (67.0–165.3)	101.1 (57.5–131.2)
Seafood	46.6 (20.0–89.6)	45.9 (18.7–94.2)	46.9 (20.2–87.8)	27.5 (13.2–53.5)	27.7 (13.2–59.9)	23.4 (15.6–48.8)	48.9 (21.7–90.3)	48.7 (21.2–96.0)	49.9 (21.7–90.0)
Canned fish	71.3 (40.0–95.0)	77.9 (49.2–95.8)	64.0 (36.8–86.1)	64.0 (26.3–86.1)	70.7 (45.6–92.5)	42.9 (21.5–79.8)	72.2 (43.4–95.0)	77.9 (51.0–97.0)	68.2 (40.3–91.2)
Fish/seafood products	64.0 (23.0–142.0)	60.0 (23.0–142.0)	64.0 (23.0–126.9)	75.0 (36.0–142.0)	60.0 (36.0–142.0)	85.5 (35.5–158.5)	62.9 (23.0–142.0)	62.0 (23.0–142.0)	62.9 (23.0–124.4)
Processed meat	44.2 (18.5–90.0)	48.0 (23.0–100.0)	37.5 (17.0–76.0)	40.9 (17.0–86.0)	45.0 (19.7–89.0)	32.3 (17.0–74.1)	45.0 (19.4–94.0)	50.0 (25.0–101.4)	38.0 (17.0–76.9)
Sausage	101.0 (89.0–178.0)	151.5 (89.0–202.0)	101.0 (89.0–178.0)	89.0 (87.0–178.0)	94.0 (89.0–178.0)	89.0 (75.0–133.5)^^^	151.5 (89.0–190.0)	174.0 (89.0–202.0)	101.0 (89.0–178.0)
Ham	17.0 (17.0–34.0)	17.4 (17.0–34.0)	17.0 (17.0–26.4)	17.0 (17.0–33.6)	17.0 (17.0–34.0)	17.0 (14.8–26.0)	17.0 (17.0–34.0)	18.5 (17.0–34.8)	17.0 (17.0–27.6)
Bacon	24.5 (13.3–48.0)	31.2 (15.2–50.0)	21.6 (10.7–43.5)	18.8 (10.0–41.1)	18.9 (11.4–43.1)	18.6 (9.6–35.3)	28.2 (14.0–48.0)	32.0 (16.0–62.7)	22.0 (11.3–44.0)
Salami	18.5 (8.9–31.4)	20.9 (11.2–37.0)	15.0 (8.1–23.3)	15.0 (6.8–23.0)	15.0 (6.1–23.0)	15.0 (7.2–23.0)	20.3 (9.4–36.0)	23.0 (14.2–4.0)	15.8 (8.3–24.5)
Luncheon meat	56.0 (45.0–104.0)	56.0 (45.0–104.0)	52.0 (45.0–104.0)	45.0 (28.0–67.0)	45.0 (28.0–56.0)	45.0 (28.0–70.5)	67.5 (45.0–104.0)	75.0 (45.0–104.0)	67.0 (45.0–104.0)
Other	45.7 (22.9–68.0)	47.6 (41.3–75.0)	45.7 (7.7–61.4)	47.6 (41.3–68.0)	45.7 (41.3–68.0)	47.6 (34.3–91.5)	45.7 (10.6–63.6)	56.7 (45.7–91.5)	41.3 (7.2–56.7)
Total Meat/poultry/fish	139.1 (80.0–219.2)	161.9 (90.7–254.9)	122.1 (71.3–191)	104.8 (59.4–175.8)	114.0 (64.2–192.1)	97.0 (55.3–163.2)	150.0 (89.0–232.0)	178.8 (104.0–273.2)	129.3 (78.2–198.0)

^^^ *P*-value <0.005 for gender difference from Whitney U test

^a^No data reported if meat type was consumed by less than 1.0% of the population

For adults, per-consumer intake of all meat types was greater for males than females (*P* < 0.01), except seafood and fish/seafood products. For children, per-consumer intake of all meat types in red meat, poultry, and fish/seafood was greater for males than females (*P* < 0.01). For children, per-consumer intake of processed meat types was similar between males and females, except sausage (males 94.0 g/day, females 89.0 g/day, *P* < 0.01). No significant differences were observed in per-consumer intakes when analyzed by socio-economic quintiles.

### Disaggregated classification results: Nutrient contribution

Meat/poultry/fish contributed 25.0% of total energy intake, 48.7% of protein intake, 48.9% of total fat intake, 25.8% of iron, and 37.9% of zinc intake (Table 5). No significant differences were observed by gender, except females reported significantly larger contribution of total fat from meat/poultry/fish than males. For children, the contribution of meat/poultry/fish to all nutrients reported increased with increasing age (*P* < 0.01), whereas for adults this decreased with advancing age (*P* < 0.01).

**Table 5:** Proportion of energy and key nutrients from meat/poultry/fish by gender and age group (2–18 years and 19+ years) per day after disaggregation of mixed dishes

Age (year)	Energy %			Protein %			Total fat %			Saturated fat %			Long-chain omega 3 fatty acids %			Iron %			Zinc %		
Male	2–18	19+	Total	2–18	19+	Total	2–18	19+	Total	2–18	19+	Total	2–18	19+	Total	2–18	19+	Total	2–18	19+	Total
Mean intake^a^	8636	9955	9655	82.0	104.6	99.4	74.3	83.7	81.5	31.4	31.5	31.5	147.1	301.9	266.8	10.8	12.6	12.2	10.2	12.6	12.0
Beef	7.8	8.7	8.1	14.5	16.7	15.2	9.6	11.5	10.3	8.9	11.3	9.9	12.8	9.1	10.0	10.2	11.8	10.8	16.8	18.7	17.3
Lamb	1.6	2.2	2.0	3.0	4.2	3.7	2.0	2.9	2.5	1.8	2.8	2.4	2.7	2.3	2.4	2.1	3.0	2.6	3.5	4.7	4.2
Pork	0.9	1.6	1.4	1.7	3.1	2.7	1.2	2.1	1.8	1.1	2.1	1.7	1.5	1.7	1.7	1.2	2.2	1.9	2.0	3.5	3.0
Kangaroo	0.0	0.1	0.1	0.0	0.1	0.1	0.0	0.1	0.1	0.0	0.1	0.1	0.0	0.1	0.1	0.0	0.1	0.1	0.1	0.1	0.1
Game	0.0	0.1	0.1	0.0	0.3	0.2	0.0	0.1	0.1	0.0	0.1	0.1	0.0	0.3	0.2	0.0	0.4	0.3	0.0	0.3	0.2
Organ/offal	0.0	0.0	0.0	0.2	0.0	0.0	0.0	0.0	0.0	0.0	0.0	0.0	0.2	0.1	0.1	0.2	0.1	0.1	0.2	0.0	0.1
Chicken	6.4	7.5	6.9	14	16.2	15.1	8.1	10.1	9.1	5.7	7.8	6.7	5.8	3.9	4.4	4.8	5.5	5.1	6.4	6.8	6.5
Fish/seafood	2.0	3.6	2.7	4.1	7.4	5.7	2.7	4.8	3.7	1.5	2.9	2.2	39.9	53.0	49.7	1.5	3	2.2	1.4	3	2.2
Processed meat	5.2	4.5	4.8	7.9	6.7	7.3	8.6	7.8	8.2	8.9	8.6	8.7	5.6	2.6	3.3	5.1	4.4	4.7	7.8	6	6.9
Total meat/poultry/fish	24.1	28.2	26.0	45.3	54.8	50.0	32.3	39.4	35.9	27.9	35.8	31.9	68.6	73.1	72.0	25.0	30.6	27.8	37.9	43.3	40.6
Female	2–18	19+	Total	2–18	19+	Total	2–18	19+	Total	2–18	19+	Total	2–18	19+	Total	2–18	19+	Total	2–18	19+	Total
Mean intake^a^	7334	7420	7402	67.8	77.9	75.7	63.8	64.2	64.1	26.6	24.0	24.6	121.5	261.3	231.2	8.6	9.7	9.4	8.0	9.4	9.1
Beef	7.3	7.4	7.2	13.4	14.6	13.6	14.7	17.8	15.9	7.9	8.9	8.2	13.3	7.2	9.9	9.5	9.3	9.1	15.2	14.9	14.7
Lamb	1.5	1.9	1.8	2.8	3.7	3.3	3.1	4.5	3.9	1.6	2.2	2.0	2.8	1.8	2.4	2.0	2.3	2.2	3.1	3.8	3.6
Pork	0.9	1.4	1.3	1.6	2.7	2.4	1.8	3.3	2.8	1.0	1.7	1.4	1.6	1.3	1.7	1.1	1.7	1.6	1.8	2.8	2.6
Kangaroo	0.0	0.1	0.1	0.0	0.1	0.1	0.0	0.1	0.1	0.0	0.1	0.1	0.0	0.1	0.1	0.0	0.1	0.1	0.1	0.1	0.1
Game	0.0	0.1	0.0	0.0	0.3	0.2	0.0	0.3	0.2	0.0	0.1	0.0	0.0	0.2	0.2	0.0	0.3	0.2	0.0	0.3	0.2
Organ/offal	0.0	0.0	0.0	0.1	0.0	0.0	0.1	0.0	0.0	0.0	0.0	0.0	0.2	0.0	0.0	0.2	0.0	0.0	0.2	0.0	0.0
Chicken	6.6	7.2	6.9	15.2	16.2	15.7	16.8	19.6	18.2	5.9	7	6.5	7.2	3.5	5.4	4.7	4.8	4.7	6.6	6.2	6.4
Fish/seafood	1.7	3.7	2.7	3.7	8.1	5.9	4.1	9.9	7	1.1	2.9	1.9	58.6	72.9	60.7	1.3	3	2.2	1.3	3.2	2.2
Processed meat	4.5	3.7	4.0	7.3	5.6	6.5	8.0	6.8	7.4	7.3	6.7	7.0	5.0	2.0	3.5	4.5	3.3	3.9	7.1	4.4	5.7
Total meat/poultry/fish	22.4	25.4	24.0	44.2	51.1	47.6	48.7	62.3	55.5	24.8	29.5	27.2	68.7	69.0	82.3	23.5	24.8	24.1	35.5	35.6	35.6

^a^Mean intake of energy in Kj, protein in gram, total fat in gram, saturated fat in gram, long-chain omega 3 fatty acids, zinc, and iron in milligram

Red meat was the largest contributor among all meat/poultry/fish categories to nutrient intakes reported, although fish/seafood was the largest contributor to long-chain omega 3 fatty acids.

These nutrient contributions reflect intakes based on the disaggregated data only. For comparative purposes, analysis using the classification prior to disaggregation is shown in Additional file 1: Table S3. Results from this analysis reveal that energy, protein, total fat, saturated fat, iron and zinc contributed from meat/poultry/fish were significantly higher for the disaggregated classification (differences before and after disaggregation: energy 345 Kj; protein 4.8 g; total fat 8.0 g; saturated fat 1.6 g, iron 0.4 mg; zinc 0.5 mg).

## Discussion

This secondary analysis provides nationally representative data on meat/poultry/fish consumption patterns using disaggregated data for over 1500 recipes and meat products. The use of disaggregated data reflect a more precise consumption profile of this food group and demonstrate the effect of disaggregation on survey data using broad food groupings. Our analysis revealed that the use of broad food groupings to estimate meat consumption tended to overestimate intakes of red meat by 9%, poultry (25%), and fish (18%) but underestimated intakes of processed meat (−17%). The overestimation of red meat, poultry and fish was likely due to the broad food grouping method capturing all food components (such as vegetables and grains) within mixed dishes, (for example curries and stir-fries), where meat/poultry/fish was a major component. The underestimation of processed meats is likely due to the broad food grouping method not capturing meats from mixed dishes where meat is only a minor component, such as pizzas, pies and pasta dishes. Consequently, nutrient contributions from meat/poultry/fish were also underestimated in non-disaggregated survey data, with estimates showing differences in key nutrient contributions up to 50%. These findings confirm results from previous studies [21, 25, 26] that overall meat/poultry/fish intake is overestimated in national dietary surveys, when disaggregation is not taken into account. Studying the effect of disaggregation on consumption data is thus a significant issue that should be considered in epidemiological studies examining associations between food/nutrient intake and health outcomes [27], for monitoring changes over time [10], and for the development of food based dietary guidelines. Hence, this study provides the detailed meat/poultry/fish classifications and stratifications for gender, age, and socio-economic subgroups.

Based on the disaggregated data, more than 90% of participants reported consuming meat/poultry/fish on the day prior to the interview, with red meat (e.g. beef, lamb, pork) consumed by approximately half, poultry and processed meat by two fifths, and fish/seafood by one fifth of the population. Per-capita data revealed total meat/poultry/fish intake was 152 g per day (118 g for children and 162 g for adults). Red meat (beef, lamb and pork) was the highest contributing meat category (38%), followed by poultry (30%), processed meat (17%) and fish/seafood (15%). Within the red meat category, beef was the most popular meat type, followed by lamb and pork while kangaroo and game meat were consumed in minimal amounts. In the poultry category, chicken was the major meat type, with other poultry meats such as duck, turkey and quail only contributing less than 2 %. In the fish/seafood category, finfish and canned fish were the highest contributors. Organ and offal meat consumption was negligible. In the processed meat category, sausage followed by ham and bacon contributed most to per-capita intake. Overall, the most popular meat type for children and adults of all ages, was chicken.

Comparison of per-capita intakes to other surveys is problematic due to methodological differences. For example, previous analyses of Australian national nutrition surveys have not disaggregated all meat components from mixed dishes as was done in the present analysis [2, 28]. Our data can be compared to similarly disaggregated data from the United Kingdom (UK) and the United States (US). In the UK, average daily intakes of meat/poultry/fish in 2006–11 were 144–173 g for males and 100–117 g for females aged between 36 and 64 years [12], compared to our findings of 193 g for adult males, and 136 g for adult females. Higher intakes were reported in the US at 255–281 g per day for adults in 2004 [29].

Red meat consumption for adult males and females was estimated at 75 g and 62 g per day, respectively. Assuming these mean intakes are representative of daily intakes, the total red meat intake on a weekly basis can be estimated to be 525 g for males and 430 g for females. The recommendation for red meat consumption for Australian adults is set at 455 g per week based on both meeting nutrient requirements and as a limit to avoid excessive consumption associated with increased risk of colorectal cancer risk (> 700 g cooked red meat per week) [7]. Although current intakes are close to the recommended intake for women and somewhat higher for men, the proportion regularly consuming excessive red meat intakes cannot be accurately determined from one or two days of recall data [30, 31].

Consumption of fish/seafood was 22 g per-capita per day, translating to 154 g per week, or approximately 1.5 serves per week. This is below the recommended dietary guidelines (two serves per week) [13] but comparable to previous Australian studies by Meyer et al. [32] and Rahmawaty et al. [33]. In Europe, the per-capita total fish/seafood intake using disaggregated data was similar for adults, a 27 g for men and 29 g for women [23] compared to our analysis (27 g for men and 24 g for women).

Consumption of processed meat was relatively common with 38% of the population reporting consuming some type of processed meat on the day prior to the interview, although per-consumer intake was lower than that for non-processed meats (45 g versus 80–100 g). Previous findings that processed pork (including ham and bacon) is the most frequently consumed type of pork in the 2007 Australian National Children’s Nutrition and Physical Activity Survey are reflected in our analysis. Comparisons with other surveys are limited due to the differences in definition of processed meats and reporting methods [34, 35].

Per-consumer data revealed that some meat types were reported in larger amounts per day than others, for example the quantities of lamb, sausages and finfish were larger than those of beef and pork. This is likely to reflect the different portion sizes of various meat cuts consumed, for example two sausages versus a small amount of minced pork in a stir-fry. Most processed meats (except sausage) and seafood were consumed in small quantities (20–50 g) per day.

Socio-economic status was positively associated with fish/seafood consumption but not with any other meat/poultry category. Previous analysis in Australia and the US showed only very modest and inconsistent differences of total meat consumption across socio-economic categories [1, 29]. Data from the U.S. national survey showed slightly larger total meat/poultry/fish consumption in higher-income men, as well as a smaller red meat consumption among high-income women [29]. This may be due to the difference in the cost of meat/poultry/fish types and people’s perceptions and response to nutrition education [5, 36–38].

The contribution of meat/poultry/fish to intakes of key nutrients was highlighted in this secondary analysis, particularly for protein, omega-3 fatty acids, iron, and zinc. Beef, chicken, and fish contributed higher amounts of these nutrients to overall intakes when compared with processed meat. Similar findings have been indicated in the U.S. for iron and zinc intake contribution from meat/poultry/fish consumption [39]. In contrast, processed meat contributed significant amounts of saturated fat intake, a consistent observation in previous national surveys [25, 28, 34]. Evidence from observational studies in Ireland and Britain reported that consumption of processed meat was associated with poorer diet quality, lower socio-economic characteristics, and other health related risk factors when compared with other categories of meat/poultry/fish [26, 40].

The significant discrepancy between the contribution of meat/poultry/fish to intakes of key nutrients, in combination with the high proportion of participants consuming meat/poultry/fish, highlights the importance of recipe disaggregation. Another area that would benefit from the use of disaggregated data is the risk assessment for chemical food contaminants that may occur in meat.

Of particular importance to nutrition surveys is a widely observed tendency for people to misreport their food intake. The prevalence of potential under-reporting behaviour in the NNPAS was calculated to be between 19 and 23% using the Goldberg cut-off (energy intake vs. basal metabolic rate < 0.9) by the ABS [16]. The impact of misreporting in the assessment of meat consumption is unknown. Some studies suggest that unhealthy foods with high fat and sugar contents are more likely to be under-reported than core foods such as meats and alternatives [41, 42] although this is not a consistent finding [43]. Further work into the impact of under-reporting on the consumption patterns of different foods from the survey results is required.

The strength of this study included the use of a large representative sample of Australian children and adults. All meat categories and meat types were well described, and stratifications by gender, age, and socio-economic subgroups were undertaken to enable comparison to other studies. A potential limitation was the use of a single 24-h recall to estimate food consumption, although this is a valid method to estimate mean group intakes. The second day was not used as the individual meat types were infrequently consumed (e.g. lamb and pork) and the statistical methods analysing usual intake of such foods requires further dietary assessment on usual frequency of consumption [30].

## Conclusions

Our analysis on the basis of recent nationally representative data shows that red meat, poultry and fish intake is over-estimated and processed meat under-estimated compared with more precise disaggregated data. These findings emphasise the need for population studies to disaggregate reported food information to provide a more precise estimate of consumption and are of particular relevance when considering associations between foods/nutrients and health outcomes [27], monitoring intakes over time, and in the development of food-based dietary guidelines.

## Additional files

Additional file 1: Table S1. Categorisation of meat/poultry/fish. **Table S2.** Mean per-capita intake of meat/poultry/fish (g) by socio-economic category after disaggregation of mixed dishes. **Table S3.** Daily energy and key nutrient intakes from meat/poultry/fish consumption – comparison before and after disaggregation of mixed dishes. **Table S4.** Proportion of persons consuming meat/poultry/fish by gender for children and adults after disaggregation of mixed dishes and per-consumer intake of meat/poultry/fish by gender for children and adults, median (25th and 75th percentile) after disaggregation of mixed dishes, g/day. (DOCX 26 kb)

## Abbreviations

ABS: Australian bureau of statistics
AGHE: Australian guidelines of healthy eating
FSANZ: Food standards Australia and New Zealand
NNPAS: 2011–12 National nutrition and physical activity survey of Australia
SEIFA: Socio-economic index of disadvantage for areas
