# Supplementary material for: Consumption patterns of meat, poultry, and fish after disaggregation of mixed dishes: secondary analysis of the Australian National Nutrition and Physical Activity Survey 2011–12
Source: BMC Nutr. 2017 Jul 1;3:52. doi: 10.1186/s40795-017-0171-1 (PMC7050704; doi:10.1186/s40795-017-0171-1)
Supplement: Supplementary file 1 — Categorisation of meat/poultry/fish. Table S2. Mean per-capita intake of meat/poultry/fish (g) by socio-economic category after disaggregation of mixed dishes. Table S3. Daily energy and key nutrient intakes from meat/poultry/fish consumption – comparison before and after disaggregation of mixed dishes. Table S4. Proportion of persons consuming meat/poultry/fish by gender for children and adults after disaggregation of mixed dishes and per-consumer intake of meat/poultry/fish by gender for children and adults, median (25th and 75th percentile) after disaggregation of mixed dishes, g/day. (DOCX 26 kb) [file 40795_2017_171_MOESM1_ESM.docx]

Additional file 1

**Table S1. Categorisation of meat/poultry/fish**

| **Category** | **Type** | **Common examples** |
| --- | --- | --- |
| **Red meat** | Beef | Beef, veal, all cuts/mince |
|  | Lamb | Lamb, mutton, all cuts/mince |
|  | Pork | Pork, all cuts/mince |
|  | Kangaroo | Kangaroo, all cuts/mince |
|  | Game meat | Goat, rabbit, all cuts |
|  |  |  |
| **Poultry** | Chicken | Chicken, all cuts/mince |
|  | Other | Duck, turkey, quail, all cuts/mince |
|  |  |  |
| **Organ/offal meat** | Offal/organ | Liver, kidney, heart |
|  |  |  |
| **Fish/seafood** | Finfish | Fish fillets, whole fish, fish pieces |
|  | Seafood | Prawns, oysters, mussels, crabs |
|  | Canned fish | Canned tuna, canned, salmon, sardines |
|  | Fish/seafood products | Smoked salmon, fish cake, seafood sticks |
|  |  |  |
| **Processed meat** | Sausage^ | Beef/pork/chicken sausage, BBQ sausage |
|  | Ham | All ham types |
|  | Bacon | All bacon types |
|  | Salami | Salami, cabanossi |
|  | Luncheon meat | Corned beef, devon, smoked turkey |
|  | Other | Frankfurters, spam, beef jerky |

^ Sausage applies to traditional Australian sausages described in the Australia New Zealand Food Standards Code Standard 2.2.1 as ‘Sausage is meat that is minced or comminuted meat or a combination thereof which may be combined with other foods encased or formed into discrete units but does not include meat formed or joined into the semblance of meat’.

**Table S2.** Mean per-capita intake of meat/poultry/fish (g) by socio-economic category after disaggregation of mixed dishes

|  | **Socio-economic category - SEIFA quintiles** | | | | |
| --- | --- | --- | --- | --- | --- |
| **Male** | **1st** | **2nd** | **3rd** | **4th** | **5th** |
| **Red meat** | **69.6** | **66.1** | **72.8** | **70.3** | **64.8** |
| Beef | 49.5 | 47.2 | 54.0 | 47.0 | 43.2 |
| Lamb | 10.2 | 10.5 | 10.4 | 13.4 | 13.6 |
| Pork | 9.3 | 8.1 | 8.2 | 9.1 | 7.5 |
| Kangaroo | 0.3 | 0.0 | 0.2 | 0.8 | 0.5 |
| Game meat | 0.3 | 0.3 | 0.1 | 0.0 | 0.1 |
| **Poultry** | **49.6** | **56.0** | **50.8** | **53.0** | **53.7** |
| **Fish/seafood^** | **19.9** | **21.2** | **19.7** | **21.0** | **25.3** |
| Fish^ | 12.5 | 12.5 | 11.2 | 12.1 | 13.2 |
| Seafood^ | 2.6 | 3.3 | 2.6 | 3.8 | 5.1 |
| Canned fish^ | 4.8 | 5.4 | 5.9 | 5.1 | 7.0 |
| **Organ/offal meat** | **0.0** | **0.0** | **0.2** | **0.0** | **0.0** |
| **Processed meat** | **39.3** | **32.4** | **37.0** | **32.6** | **33.7** |
| **Total meat/poultry/fish** | **178.4** | **175.7** | **180.4** | **177.0** | **177.6** |
| **Female** | **1st** | **2nd** | **3rd** | **4th** | **5th** |
| **Red meat** | 44.6 | 52.4 | 46.7 | 47.1 | 44.0 |
| Beef | 30.9 | 37.5 | 33.5 | 32.1 | 31.0 |
| Lamb | 6.7 | 8.5 | 6.5 | 8.7 | 8.3 |
| Pork | 6.6 | 5.9 | 6.0 | 6.2 | 4.7 |
| Kangaroo | 0.0 | 0.5 | 0.6 | 0.0 | 0.1 |
| Game meat | 0.2 | 0.0 | 0.1 | 0.1 | 0.0 |
| **Poultry** | **41.3** | **36.8** | **42.7** | **39.5** | **41.8** |
| **Fish/seafood^** | **16.8** | **18.5** | **20.5** | **18.2** | **21.5** |
| Fish^ | 9.1 | 9.9 | 9.8 | 8.5 | 10.9 |
| Seafood | 3.4 | 3.2 | 4.0 | 3.5 | 3.5 |
| Canned fish^ | 4.3 | 5.4 | 6.7 | 6.2 | 7.1 |
| **Organ/offal meat** | **0.1** | **0.0** | **0.0** | **0.0** | **0.0** |
| **Processed meat** | **22.9** | **20.9** | **22.9** | **26.0** | **20.5** |
| **Total Meat/poultry/fish** | **125.6** | **128.7** | **132.9** | **130.7** | **127.9** |

^P-value <0.05 for different across SEIFA category from Analysis of Variance

**Table S3.** Daily energy and key nutrient intakes from meat/poultry/fish consumption – comparison before and after disaggregation of mixed dishes

|  | Before disaggregation*^+^* | After disaggregation ^§^ | Difference  (%) |
| --- | --- | --- | --- |
| **Red meat** |  |  |  |
| Energy (Kj) | 586 | 931 | -37.0* |
| Protein (g) | 13.2 | 18.0 | -26.7* |
| Total fat (g) | 5.8 | 13.8 | -58.0* |
| Monounsaturated fat (g) | 2.6 | 6.9 | -27.7* |
| Polyunsaturated fat (g) | 0.6 | 3.3 | -10.9* |
| Saturated fat (g) | 2.0 | 3.6 | -44.4* |
| Long-chain omega 3 fatty acids (mg) | 36.1 | 41.1 | -12.2* |
| Iron (mg) | 1.1 | 1.5 | -26.7* |
| Zinc (mg) | 2.2 | 2.7 | -18.5* |
| **Poultry** |  |  |  |
| Energy (Kj) | 457 | 584 | -21.7* |
| Protein (g) | 11.6 | 13.4 | -13.4* |
| Total fat (g) | 5.4 | 10.1 | -46.5* |
| Monounsaturated fat (g) | 2.5 | 4.7 | -46.8* |
| Polyunsaturated fat (g) | 0.9 | 3.6 | -69.2* |
| Saturated fat (g) | 1.6 | 1.8 | -11.1* |
| Long-chain omega 3 fatty acids (mg) | 11.0 | 12.8 | -14.1* |
| Iron (mg) | 0.4 | 0.5 | -20.0* |
| Zinc (mg) | 0.6 | 0.7 | -14.3* |
| **Fish/seafood** |  |  |  |
| Energy (Kj) | 197 | 228 | -13.7* |
| Protein (g) | 5.2 | 5.0 | 4.0 |
| Total fat (g) | 2.4 | 3.9 | -38.5* |
| Monounsaturated fat (g) | 1.0 | 2.1 | -52.3* |
| Polyunsaturated fat (g) | 0.7 | 1.2 | -41.7* |
| Saturated fat (g) | 0.5 | 0.6 | -16.7* |
| Long-chain omega 3 fatty acids (mg) | 138.6 | 131.5 | 5.4 |
| Iron (mg) | 0.2 | 0.2 | 0.0 |
| Zinc (mg) | 0.3 | 0.3 | 0.0 |
| **Processed meat** |  |  |  |
| Energy (Kj) | 205 | 370 | -44.7* |
| Protein (g) | 5.0 | 6.0 | -16.7* |
| Total fat (g) | 3.4 | 5.6 | -39.3* |
| Monounsaturated fat (g) | 1.5 | 2.9 | -48.3* |
| Polyunsaturated fat (g) | 0.2 | 0.5 | -60* |
| Saturated fat (g) | 1.4 | 2.2 | -36.4* |
| Long-chain omega 3 fatty acids (mg) | 11.4 | 14.1 | -19.1* |
| Iron (mg) | 0.3 | 0.5 | -40.0* |
| Zinc (mg) | 0.5 | 0.6 | -16.7* |
| **Total meat/poultry/fish** |  |  |  |
| Energy (Kj) | 1445 | 2114 | -31.6* |
| Protein (g) | 35.0 | 42.4 | -17.5* |
| Total fat (g) | 17.0 | 33.4 | -49.1* |
| Monounsaturated fat (g) | 7.6 | 16.6 | -54.2* |
| Polyunsaturated fat (g) | 2.4 | 8.6 | -72.1* |
| Saturated fat (g) | 5.5 | 8.2 | -32.9* |
| Long-chain omega 3 fatty acids (mg) | 197.1 | 199.5 | -1.2 |
| Iron (mg) | 2.0 | 2.7 | -25.9* |
| Zinc (mg) | 3.6 | 4.3 | -16.3* |

* P-value < 0.05 from independent t-test

^+^Values refer to the mass of all individually recorded items, and the total mass of mixed dishes where meat/poultry/fish was a major component but excludes mass from dishes where meat/poultry/fish was a minor component

^§^Values refer to the mass of the meat/poultry/fish components from all individually recorded items and from mixed dishes where meat/poultry/fish was a major or minor component

**Table S4:** Proportion of persons consuming meat/poultry/fish by gender for children and adults after disaggregation of mixed dishes and per-consumer intake of meat/poultry/fish by gender for children and adults, median (25^th^ and 75^th^ percentile) after disaggregation of mixed dishes, g/day*

|  | Proportion (%) | | | | | Per – consumer intake  Median (25th – 75^th^ percentile)* | | | | |
| --- | --- | --- | --- | --- | --- | --- | --- | --- | --- | --- |
|  | Total | Children | Adults | Male | Female | Total | Children | Adults | Male | Female |
| Red meat | 48.6 | 46.0 | 49.4 | 52.4 | 45.2^ | 98.1 (47.5-163.5) | 70.8 (34.7-131.2) | 104.0 (54.0-169.0) | 107.0 (52.3-181.9) | 91.0 (43.8-150.0) |
| Beef | 38.0 | 38.3 | 38.0 | 41.8 | 34.7^ | 83.3 (36.3-155.3) | 62.8 (28.4-116) | 94.2 (40.8-164.5) | 93.8 (40.8-166.2) | 76.5 (33.2-146) |
| Lamb | 8.1 | 6.1 | 8.7 | 8.6 | 7.6^ | 104.0 (61.0-156.0) | 96.5 (44.5-155.8) | 104.0 (65.1-156.0) | 124.0 (68.6-171.7) | 95.5 (52.8-142) |
| Pork | 7.5 | 5.6 | 8.0 | 8.0 | 7.0 | 75. (30.5-120.0) | 51.7 (23.6-107.4) | 82.0 (35.4-125.0) | 80.0 (36.0-148.0) | 73.8 (24.5-109.2) |
| Kangaroo | 0.3 | 0.4 | 0.3 | 0.4 | 0.3 |  |  |  |  |  |
| Game meat | 0.1 | 0.0 | 0.1 | 0.1 | 0.1 |  |  |  |  |  |
| Poultry | 37.7 | 38.4 | 37.5 | 37.7 | 37.7 | 95.0 (57.0-166.0) | 80.0 (46.8-129.8) | 100.0 (60.0-176.2) | 108.0 (60.3-184.2) | 86.6 (51.2-143) |
| Chicken | 36.8 | 38.0 | 36.5 | 36.8 | 36.9 | 93.6 (57.0-162.7) | 80.0 (46.8-129.5) | 100.0 (59.8-175.2) | 107.5 (60.3-184.2) | 85.9 (50.9-142.5) |
| Other | 1.3 | 0.7 | 1.4 | 1.4 | 1.1 | 92.3 (33.6-151.9) | 63.6 (27.7-150.0) | 93.0 (36.0-153.8) | 98.0 (70.4-166.0) | 80.1 (23.1-143) |
| Organ/offal meat | 0.1 | 0.1 | 0.1 | 0.2 | 0.1 |  |  |  |  |  |
| Fish/seafood | 21.4 | 14.3 | 23.5 | 20.3 | 22.4^ | 81.9 (46.1-126.8) | 61.5 (30.0-101.3) | 86.1 (49.0-133.3) | 92.5 (51.4-144.0) | 77.5 (41.6-117.3) |
| Finfish | 9.7 | 7.1 | 10.4 | 10.0 | 9.4 | 103.5 (53.7-136.8) | 62.7 (38.7-107.0) | 104.5 (62.4-150.0) | 106.4 (62.4-156.2) | 92.5 (46.5-123.1) |
| Seafood | 5.4 | 3.1 | 6.1 | 5.1 | 5.7 | 46.6 (20.0-89.6) | 27.5 (13.2-53.5) | 48.9 (21.7-90.3) | 45.9 (18.7-94.2) | 46.9 (20.2-87.8) |
| Canned fish | 7.8 | 4.6 | 8.8 | 7.1 | 8.5^ | 71.3 (40.0-95.0) | 64.0 (26.3-86.1) | 72.2 (43.4-95.0) | 77.9 (49.2-95.8) | 64.0 (36.8-86.1) |
| Fish/seafood products | 1.6 | 0.7 | 1.8 | 1.4 | 1.7 | 64.0 (23.0-142.0) | 75.0 (36.0 -142.0) | 62.9 (23.0-142.0) | 60.0 (23.0-142.0) | 64.0 (23.0-126.9) |
| Processed meat | 37.8 | 42.9 | 36.2 | 41.4 | 34.6^ | 44.2 (18.5-90.0) | 40.9 (17.0-86.0) | 45.0 (19.4-94.0) | 48.0 (23.0-100.0) | 37.5 (17.0-76.0) |
| Sausage | 7.1 | 9.0 | 6.5 | 8.5 | 5.8^ | 101.0 (89.0-178.0) | 89.0 (87.0-178.0) | 151.5 (89.0-190.0) | 151.5 (89.0-202.0) | 101.0 (89.0-178.0) |
| Ham | 18.0 | 21.1 | 17.1 | 19.4 | 16.8^ | 17.0 (17.0-34.0) | 17.0 (17.0-33.6) | 17.0 (17.0-34.0) | 17.4 (17.0-34.0) | 17.0 (17.0-26.4) |
| Bacon | 13.8 | 14.3 | 13.6 | 15.3 | 12.4^ | 24.5 (13.3-48.0) | 18.8 (10.0-41.1) | 28.2 (14.0-48.0) | 31.2 (15.2-50.0) | 21.6 (10.7-43.5) |
| Salami | 5.7 | 6.3 | 5.5 | 6.7 | 4.8^ | 18.5 (8.9-31.4) | 15.0 (6.8-23.0) | 20.3 (9.4-36.0) | 20.9 (11.2-37.0) | 15.0 (8.1-23.3) |
| Luncheon meat | 3.7 | 4.0 | 3.7 | 4.2 | 3.4 | 56.0 (45.0-104.0) | 45.0 (28.0-67.0) | 67.5 (45.0-104.0) | 56.0 (45.0-104.0) | 52.0 (45.0-104.0) |
| Other | 1.9 | 3.3 | 1.4 | 2.2 | 1.5 | 45.7 (22.9-68.0) | 47.6 (41.3-68.0) | 45.7 (10.6-63.6) | 47.6 (41.3-75.0) | 45.7 (7.7-61.4) |
| Total meat/poultry/fish | 91.3 | 90.4 | 91.5 | 92.6 | 90.1^ | 139.1 (80.0-219.2) | 104.8 (59.4-175.8) | 150.0 (89.0-232.0) | 161.9 (90.7-254.9) | 122.1 (71.3-191) |

^ P-value <0.05 for gender difference from Chi-square test

* No data reported if meat type was consumed by less than 1.0% of the population
